# A systematic review exploring the association between the human gut microbiota and brain connectivity in health and disease

**DOI:** 10.1038/s41380-023-02146-4

**Published:** 2023-07-21

**Authors:** Danique Mulder, Esther Aarts, Alejandro Arias Vasquez, Mirjam Bloemendaal

**Affiliations:** 1 Department of Psychiatry, Radboud University Medical Center, Donders Institute for Brain, Cognition and Behaviour, Geert Grooteplein Zuid 10, 6525 GA Nijmegen, The Netherlands; 2 Donders Institute for Brain, Cognition and Behaviour, Radboud University, Nijmegen, The Netherlands; 3 Department of Human Genetics, Radboud University Medical Center, Donders Institute for Brain, Cognition and Behaviour, Nijmegen, The Netherlands

**Keywords:** Neuroscience, Psychiatric disorders, Biological techniques

## Abstract

A body of pre-clinical evidence shows how the gut microbiota influence brain functioning, including brain connectivity. Linking measures of brain connectivity to the gut microbiota can provide important mechanistic insights into the bi-directional gut-brain communication. In this systematic review, we therefore synthesized the available literature assessing this association, evaluating the degree of consistency in microbiota-connectivity associations. Following the PRISMA guidelines, a PubMed search was conducted, including studies published up to September 1, 2022. We identified 16 studies that met the inclusion criteria. Several bacterial genera, including *Prevotella*, *Bacteroides*, *Ruminococcus*, *Blautia*, and *Collinsella* were most frequently reported in association with brain connectivity. Additionally, connectivity of the salience (specifically the insula and anterior cingulate cortex), default mode, and frontoparietal networks were most frequently associated with the gut microbiota, both in terms of microbial diversity and composition. There was no discernible pattern in the association between microbiota and brain connectivity. Altogether, based on our synthesis, there is evidence for an association between the gut microbiota and brain connectivity. However, many findings were poorly replicated across studies, and the specificity of the association is yet unclear. The current studies show substantial inter-study heterogeneity in methodology and reporting, limiting the robustness and reproducibility of the findings and emphasizing the need to harmonize methodological approaches. To enhance comparability and replicability, future research should focus on further standardizing processing pipelines and employing data-driven multivariate analysis strategies.

## Introduction

Neuronal connections are determined by the distance between neurons. Spatially closer neurons have a higher probability of being connected than those further away. As a result, the structural wiring of the brain represents a complex network with clusters of highly connected regions (i.e., structural connectivity) [1] that provide a basis for functional communication between brain areas (i.e., functional connectivity) as measured by the temporal coincidence of neuronal activation patterns of anatomically separated brain regions [2]. Patterns of communication between fixed sets of brain regions form functional networks [3, 4], which can be identified both during active cognitive tasks and during rest.

Functional networks measured during rest (resting-state networks) reflect, among others, processes related to cognitive functions. For example, the salience network (SN) is involved in the detection of behaviorally relevant stimuli [5], the frontoparietal network (FPN) is involved in the coordination of cognitive control [6], and the default mode network (DMN) is linked to basal, stimulus-independent cognitive processes such as information integration and mind-wandering [7]. In addition to cognitive functioning, connectivity may also reflect intrinsic processes, such as emotional and interoceptive awareness [8, 9]. Dysfunction in connectivity networks is observed in a range of psychiatric and neurodevelopmental disorders, including Attention Deficit/Hyperactivity Disorder [10] and Schizophrenia [11], in certain cases even prior to diagnosis [12].

The functional and structural connectivity patterns of the brain are affected by numerous genetic and non-genetic interacting factors. Our genetic makeup has a significant biological effect on both brain structure and function: heritability studies have shown that the additive genetic contribution explains approximately 50% to 93% of the variance in structural connectivity [13] and 20% to 40% of the variance in functional connectivity [14]. Likewise, the brain can change under the influence of environmental factors. Multiple studies have reported changes in connectivity strength following a mindfulness training, both in structural [15] and functional connectivity [16]. Additionally, a systematic review concluded that a lower quality diet was related to decreased structural and functional connectivity of default mode, sensorimotor and attention networks [17].

Such environmental factors, including diet, may exert their influence on the brain through the gut-brain axis (GBA), among others via modulation of the gut microbiota [18]. The GBA refers to the bidirectional communication system connecting the gastrointestinal system with the central nervous system (CNS) through endocrine, immune, and neural/vagal pathways [19]. The gut microbiota, comprising the trillions of microbes (predominantly bacteria) residing in the intestines, can modulate gut-brain communication, for example through the production of neuroactive metabolites, and by affecting the integrity of the gastro-intestinal and blood-brain barriers [19, 20].

A majority of the studies investigating the (microbiota-)gut-brain axis (MGBA) in humans focus on behavioral measures, including clinical diagnoses and questionnaires, providing evidence for a link between the gut microbiota composition and cognitive and emotional functioning [21–23]. In recent years the number of studies incorporating neuroimaging into the microbiota-gut-brain investigation has also increased rapidly. Specifically, the acquisition of functional and structural connectivity data is relatively standardized and often done at rest (i.e., without task instructions), making it a suitable research method for many participant populations, including children and patients. Linking such connectivity measures to the gut microbiota can provide important mechanistic insights into the bi-directional gut-brain communication. Therefore, we herein systematically review the available studies associating the gut microbiota with brain connectivity, in an effort to evaluate the degree of consistency in this association.

## Method

### Search strategy

**Fig. 1:**
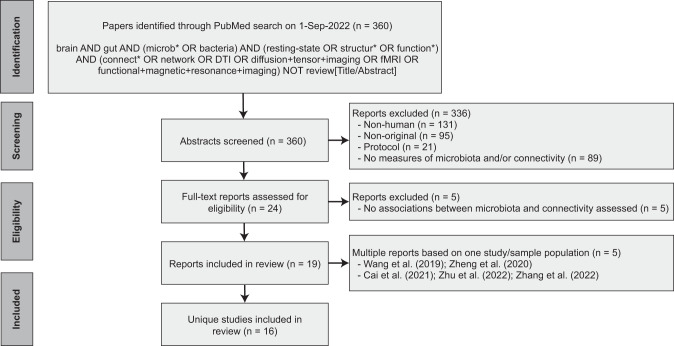
PRISMA flow diagram. PRISMA flow diagram detailing the database search, number of reports screened and number of studies included.

Following PRISMA guidelines, a systematic search on the PubMed database was conducted for reports published up to September 1, 2022. The aim was to capture all human studies that 1) collected a fecal sample to assess the gut microbiota, 2) assessed in vivo functional or structural brain connectivity, and 3) performed statistical analysis on the association between the gut microbiota and brain connectivity. Only peer-reviewed original research studies (i.e., reporting original data, analyses, and findings) published in English were included. Two independent raters (DM, MB) reviewed the titles and abstracts and came to a consensus about study inclusion. After inclusion, the following data was independently extracted by two authors (DM, MB): demographics, sample characteristics, method of gut microbiota estimation, method of brain connectivity estimation, statistical methods, and relevant results. Details on the search strategy and study inclusion are provided in Fig. 1. The PRISMA checklist is available in the Supplementary Materials.

### Consistency of the findings

The consistency of the findings was assessed on two levels. First, we looked at the gut microbiota and brain connectivity individually. Second, we assessed the specificity of the microbiota-connectivity association. For the first, the assessment was done by dividing the number of studies reporting a statistically significant association for a diversity index/genus abundance/brain network by the total number of studies assessing it. For the second, the assessment was done by counting the number of studies reporting a statistically significant microbiota-connectivity association and dividing it by the total number of studies that could have identified this association. Only findings assessed in at least three studies were interpreted. A finding was considered consistent if it was reported in at least 50% of the studies (if assessed in four or more studies), with an absolute minimum of two studies (if assessed in three studies).

### Quality assessment

Two authors (DM, MB) assessed the risk of bias in the included studies using the National Institutes of Health (NIH) National Health, Lung and Blood Institute Study Quality Assessment Tool for observational Cohort and Cross-sectional Studies [24]. The tool was modified to also be suitable for case-control and before-after studies with no control group (specified in Supplementary Table 3). We used the method section of the STORMS checklist (v1.03) [25] to assess gut microbiota measurements, and an adaptation of the guidelines published by Poldrack et al. [26] to assess brain connectivity measurements (Supplementary Table 4-5). Study quality was rated as ‘Good’ for assessments of 75% or higher; ‘Fair’ for assessments between 50% and 75%; or ‘Poor’ for assessments lower than 50%.

## Results

A comprehensive literature search on PubMed yielded 360 reports, of which a total of 19 publications based on 16 unique studies met the inclusion criteria (Fig. 1). Two of the included studies produced more than one publication ([27–29] and [30, 31]). For those studies, the findings reported in the individual publications were pooled.

**Table 1:** Overview of population characteristics.

	Ref	First author (year)	Country	Target population	Sample size	% female	Age in years (SD)	Antibiotic use	Pre-/ pro-/ synbiotic use	Quality Rating	Other
Healthy population *Adults*	[32]	Curtis (2019)	USA	Healthy (smokers/eCig users/non-smokers)	30^a^	7%	33 (2.7)			Poor	Smoking groups pooled in analyses
	[27–29]	Cai (2021)/Zhu (2022)/Zhang (2022)	China	Healthy	157	49%	22.3 (2.4)	No antibiotics month before inclusion		Fair	
	[33]	Hall (2021)	Australia	Healthy	38%	60.5%	31.7 (9.7)	No antibiotics 3 months before inclusion	No probiotics 3 months before inclusion	Fair	
	[34]	Tillisch (2017)	USA	Healthy	40	100%	28.9 (9.9)	No antibiotics month before inclusion	No probiotics month before inclusion	Fair	
	[37]	Kohn (2021)	Netherlands	Healthy	58	100%	21.5 (0.5)	No antibiotics (no period specified)		Fair	
Healthy population *Children*	[35]	Gao (2019)	USA	Infants	39	59%	1.11 (0.10)			Poor	
	[36]	Kelsey (2021)	USA	Newborns	63	41%	0.07 (0.03)			Good	
Disease population *Adults*	[44]	Dong (2020)	USA	(Obese) patients undergoing LSG	14^a^	100%	37.4 (9.7)	No antibiotics month before inclusion	No probiotics month before inclusion	Fair	Pre- and post-surgery timepoints pooled in analyses
	[42]	Strandwitz (2019)	USA	MDD	23	65%	[19–65] (min-max)			Fair	
	[30, 31]	Wang (2019)/Zheng (2020)	China	ESRD/healthy	28/19	54%/37%	43.9 (13.8)/44.1 (10.0)	No antibiotics 2 weeks before inclusion	No probiotics (no period specified)	Poor	
	[38]	Li (2022)	China	BP/healthy	44/37	50%/54%	22.0 (8.2)/21.1 (1.9)	No antibiotics 4 weeks before inclusion	No pre-/probiotics 4 weeks before inclusion	Fair	
	[43]	Dong (2022)	USA	Obese/healthy	81/216	72.8%/64.4%	32 [26-41.5]/26 [21–34]	No antibiotics 3 months before inclusion		Fair	
	[41]	Labus (2019)	Sweden	IBS/Healthy	65/21	71%	33.3 (10.2)/33.3 (9.6)	No antibiotics month before inclusion	No probiotics month before inclusion	Fair	
	[45]	Hong (2021)	China	(Obese) patients undergoing VSG	16^a^	67.0%	28.6 (4.1)	No antibiotics 3 months before inclusion	No probiotics (incl. foods) 7 days before sample collection	Good	
	[40]	Jacobs (2021)	USA	IBS patients responding to CBT/not responding to CBT	22/12	81.8%/83.3%	43.7 (13.1)/48.0 (14.9)	No antibiotics 12 weeks before inclusion		Good	
	[39]	Ahluwalia (2016)	USA	Cirrhosis (with/without HE)	74^a^	41%	54.2 (11.6)/56.2 (13.5)	No antibiotics 6 weeks before inclusion		Poor	HE groups pooled in analyses

*BP* bipolar disorder, *CBT* cognitive behavioral therapy, *ESRD* end-stage renal disease, *HE* hepatic encephalopathy, *IBS* irritable bowel syndrome, *LSG* laparoscopic sleeve gastrectomy, *MDD* major depressive disorder, *VSG* vertical sleeve gastrectomy.

^a^The sample size refers to the total number of participants, as participants in different groups/data from different timepoints were pooled for the association analysis.

### Population characteristics (Table 1)

There was a wide variation in the target populations. Eight studies were conducted in healthy individuals [27–29, 32–37] including a study conducted in smokers [32], in newborns [36], and in infants [35]. The other eight studies were performed in a range of disease populations, including bipolar disorder [38], cirrhosis [39], end stage renal disease [30, 31], irritable bowel syndrome [40, 41], major depressive disorder [42], obesity [43] and patients undergoing a laparoscopic/vertical sleeve gastrectomy [44, 45]. Four of those studies were case-controlled [30, 31, 38, 41, 43] and three were longitudinal [40, 44, 45]. All other studies performed associations between gut microbiota composition and brain connectivity based on a single group and timepoint. Further characteristics of the study populations are listed in Table 1.

**Table 2:** Overview of methodological characteristics.

	Ref	First author (year)	Microbiota analysis	Alpha diversity/ richness	Beta diversity	Taxonomic selection	Neuroimaging	Network/ROI selection	Computation of connectivity	Microbiota - connectivity statistics	Other
Healthy population *Adults*	[32]	Curtis (2019)	Collection: fresh Sequencing type: 16s rRNA sequencing Sequencing technology: Illumina Miseq Region: V4 Pipeline: UPARSE/USEARCH (v7.0.1090) Database: Silva (no version) Clustering: OTU Rarified: yes Filtering: no Transformation: relative abundance for compositional analysis	Richness: number of OTUs Diversity: Shannon	weighted UniFrac distance	Genera *Prevotella* and *Bacteroides*, based on reported alteration of the abundance of these genera in smokers	rs-fMRI Eyes open	> Primary: middle insula, based on role in interoceptive sensations > Secondary: anterior and inferior insula, ACC, habenula and striatum, based on role in data integration/reward processing	Seed-to-voxel Fisher’s z-transformed correlations with primary and secondary ROIs as seeds	Multiple linear regression, predicting connectivity using Shannon, number of OTUs, wUF component 1-2, *Prevotella* and *Bacteroides* relative abundance Covariates: smoking status Multiple testing correction: Bonferroni for primary analyses, none for secondary analyses	
	[27–29﻿]	Cai (2021)(a)/Zhu (2022)(b)/Zheng (2022) (c) (three publications based on the same study)	Collection: fresh Sequencing type: 16s rRNA sequencing Sequencing technology: Illumina Hiseq Region: V4 Pipeline: UPARSE/USEARCH (v9.1.13) Database: RDP classifier (v2.2) with GreenGenes (v201305) as training database Clustering: OTU Rarified: no Filtering: no Transformation: NA	Richness (a,b): Sobs, Ace, Chao Diversity: Shannon, Simpson		*Bacteroides*-, *Prevotella*- and *Ruminococcus*-high clusters, based on Jensen-Shannon distance and PAM	rs-fMRI Eyes closed DTI (b,c)	(a): Anterior/ posterior DMN, ECN, left/right FPN, SN, DAN, VAN, dorsal/ventral SMN, AN, lateral/posterior VN, based on ICA (b) Data-driven selection based on outcomes multi-set canonical association analysis plus joint ICA (c) No a priori ROI selection	(a) Internetwork: network-to-network Fisher’s z-transformed correlations Intranetwork: contribution of the time course to each voxel comprising an independent component (b) Mixing coefficients from multiset canonical association analysis plus joint ICA, with as input: > FC: FCD computed as number of voxel-to-voxel correlations of a particular voxel to all other voxels with r > 0.25, normalized by the global mean FCD > SC: voxelwise FA > Other: Regional homogeneity, cerebral blood flow, gray matter volume (c): SC computed as the number of fibers between each set of ROIs based on fiber tracking algorithm. FC computed as Fisher z-transformed correction between all ROIs, resulting in two 90×90 matrices > For SC and FC: computation of network topological metrics from ROI matrices as measure of connectivity ((normalized) clustering coefficient, (normalized) characteristic path length, small-worldness property, nodal degree centrality, nodal efficiency, nodal betweenness > SC-FC coupling: Pearson correlation between SC and FC ROI matrices	> Partial correlations (alpha diversity) and ANOVA (clusters) to test the association with network connectivity/FCD/FA > For significant associations: mediation analysis between alpha diversity/clusters and cognition, with connectivity as mediator Covariates: frame-wise displacement (a,c), age and sex Multiple testing correction: FDR, cluster-level FWE (b,c) or Bonferroni (c)	> Letter 3-back task to assess working memory > Digit span task to assess attention > Go/No-Go task to assess response inhibition
	[33]	Hall (2021)	Collection: Norgen’s Stool Nucleic Acid Collection and Preservation Tubes Sequencing type: 16s rRNA sequencing Sequencing technology: Illumina Miseq Region: V3-V4 Pipeline: QIIME (v1.9.1) Database: GreenGenes (v13.8) Clustering: OTU Rarified: yes (diversity analysis) Filtering: OTUs with ≥2 reads Transformation: TSS normalization and square root transformation	Diversity: (Inverse) Simpson		> Genera *Bacteroides*, *Prevotella*, *Oscillospira* and *Ruminococcus* based on clustering analysis > Bacteroidetes/ Firmicutes (B/F) ratio	Task-based fMRI	dACC and anterior insula, based on involvement in threat processing	Directed functional connectivity, computed using a computational framework (DCM), separately for threat acquisition and reversal	> Parametric Empirical Bayes (PEB) analysis to test association between Simpson, B/F-ratio and connectivity > Multiple regression to test association between abundances and connectivity (covariates: age, sex) > sCCA to test multivariate associations between abundances and connectivity Multiple testing correction: none	fMRI task: threat processing task, 2 phases: > Threat acquisition: visual stimulus A is conditioned to aversive auditory stimulus > Threat reversal: conditioning is reversed by presenting visual stimulus B with aversive stimulus, but not visual stimulus A
	[34]	Tillisch (2017)	Collection: fresh Sequencing type: 16s rRNA sequencing Sequencing technology: Roche 454 sequencing Region: V5-V6 Pipeline: QIIME (v.17) Database: GreenGenes (v201108) Clustering: OTU Rarified: no Filtering: no Transformation: relative abundance			> *Bacteroides*- and *Prevotella*-high clusters, based on weighted UniFrac > *Bacteroides* and *Prevotella* relative abundance	DTI		Relative fiber density (number of fiber tracts intersecting a region normalized by total number of fiber tracts) based on output of continuous tracking algorithm	> PLS-DA to find structural connections that can discriminate between clusters (covariates: age, total gray matter volume) > Correlation analyses between abundance and connectivity Multiple testing correction: none	
	[37]	Kohn (2021)	Collection: OMNIgene Gut Sequencing type:16s rRNA sequencing Sequencing technology: Illumina Hiseq Region: V4 Pipeline: NG-Tax (no version) Database: Silva (v128) Clustering: OTU Rarified: no Filtering: genera with prevalence ≥30% Transformation: relative abundance			No selection for compositional analyses	rs-fMRI Eyes open	left/right FPN, ECN, DMN, based on importance in neuroimaging	Dual regression to generate individual spatial maps of each network; linear modeling to obtain temporal dynamics for each network	Linked ICA to test for multivariate associative patterns of abundances and network connectivity Multiple testing correction: NA	
Healthy population *Children*	[35]	Gao (2019)	Collection: in tube with Allprotect Tissue Reagent Sequencing type: 16s rRNA sequencing Sequencing technology: Illumina Miseq Region: V1-V2 Pipeline: QIIME (no version) Database: NA, de novo OTU picking Clustering: OTU Rarified: no Filtering: NA Transformation: NA	Richness: number of OTUs, Chao Diversity: Shannon, Faith’s PD			rs-fMRI Measured during sleep	> Primary: amygdalae, based on earlier findings of involvement in gut-brain interactions > Secondary: VN, DMN, SMN, AN, SN, left/right FPN (seed placed at network maxima)	Seed-to-voxel Fisher’s z-transformed correlations	ANCOVA, predicting connectivity using diversity. Covariates: older sibling, paternal ethnicity, birth weight, postnatal age at scan, sex, twin status, parental education, residual frame-wise displacement Multiple testing correction: cluster correction	
	[36]	Kelsey (2021)	Collection: fresh Sequencing type: shotgun metagenomics Sequencing technology: Illumina Novaseq 6000 Pipeline: JAMSalpha (v1.39) Database: custom build based on NCBI GenBank Filtering: not specified Transformation: TSS normalization and square root transformation for Maaslin2. Not specified for LefSE	Richness: Chao Diversity: Shannon (taxonomic and functional: virulence factors, resistome, gene ontology terms)		No selection for compositional analyses	rs-fNIRS Measured during presentation of non-social video	left/right FPN, left/right DMN, homologous interhemispheric network, non-functional control network, based associations with internalizing disorders in adults	Average channel-to-channel Fisher’s z-transformed correlations between subset of channels corresponding to network	> Univariate regression, predicting connectivity using diversity > LefSE (low/high connectivity using median split) and Maaslin2 to identify associations with between abundances and connectivity. > For significant associations: mediation analyses between alpha diversity and behavioral temperament, with connectivity as a mediator. Covariates: determined based on significant correlation with predictor variables Multiple testing correction: FDR	Infant Behavior Questionnaire Revised to measure negative emotionality, regulation/orienting and surgency/positive emotionality
Disease population *﻿Adults*	[44]	Dong (2020)	Collection: not reported Sequencing type: 16s rRNA sequencing Sequencing technology: Illumina Hiseq Region: V4 Pipeline: dada2 in R, then QIIME2 (v2019.10) Database: Silva (v132) Clustering: ASV Rarified: yes (diversity analysis) Filtering: ASVs with prevalence >15% Transformation: relative abundances (sPLS-DA) and none (DESEq2)	Diversity: Shannon		No selection for compositional analyses	rs-fMRI Eyes not reported	Precuneus and putamen, based on changes in connectivity strength from pre- to post surgery	Fisher’s z-transformed correlation	> sPLS-DA to find ASVs that can discriminate between high/low precuneus-putamen connectivity (median split). > DESEq2 to test for group differences between high/low precuneus-putamen connectivity (covariate: time) > ANOVA to predict connectivity using Shannon Multiple testing correction: FDR	Measures collected pre- and 6 months post-surgery
	[42]	Strandwitz (2019)	Collection: fresh Sequencing type: 16s rRNA sequencing Sequencing technology: AB 3730xl DNA Analyzers Region: V4 Pipeline: QIIME (v 1.8.0) Database: not specified Clustering: OTU Rarified: no Filtering: no Transformation: relative abundance			Genus *Bacteroides*, and species *Evtepia* gabavorous (KLE1738) based on GABA producing properties as determined in culturing experiments by the authors	rs-fMRI Scanner changed during data collection (trio > prisma)	dlPFC and DMN, based on reports of altered activity in MDD	Seed-to-voxel correlations between dlPFC and all voxels within DMN + I13:I14	> ANCOVA to predict connectivity using abundance Covariates: age, sex, head motion, scanner (trio/prisma) Multiple testing correction: Monte Carlo simulation to determine the required cluster size (voxels)	
	[30, 31]	Wang (2019) (a)/Zheng (2020) (b) (two publications based on the same study)	Collection: fresh Sequencing type: 16s rRNA sequencing Sequencing technology: Illumina Miseq Region: V3-V4 Pipeline: QIIME (v1.9.1) Database: Silva (v132) Clustering: OTU Rarified: no Filtering: not specified Transformation: not specified	Diversity: Shannon		> No selection for compositional analyses > *Bacteroides*- and *Prevotella*-mainly clusters, based on LefSE (b)	rs-fMRI Eyes closed	> DMN (ICs and individual nodes), based on consistency and possible involvement in neuropsychological disorders (a) > Amygdalae, based on involvement in mood regulation (b)	> DMN: correlation between posterior and anterior DMN (ICA-based) and network topological indices ((normalized) clustering coefficient, (normalized) path length, small-worldness, global efficiency, local efficiency, nodal degree, nodal efficiency, nodal betweenness) (a) > Amygdalae: seed-to-voxel Fisher’s z-transformed correlations, between amygdalae and all other voxels in the brain (b)	> Pearson or Spearman correlation to test association between abundance and connectivity (only for genera with significant ESDR-HC group differences, only performed in cases) > Two sample t-test for assessing connectivity differences between microbial clusters (b) > For significant associations: mediation analysis between abundances and connectivity, with inflammation factors as mediator Covariates: age, sex (a,b), BMI, total brain volume (b) Multiple testing correction: FDR (a), not specified (b)	
	[38]	Li (2022)	Collection: fresh Sequencing type: shotgun metagenomics Sequencing technology: Illumina HiSeq Pipeline: not reported Database: UHGG and KEGG (no versions) Filtering: not specified Transformation: no			No selection for compositional analyses	rs-fMRI Eyes open		> Region-to-region Fisher’s z-transformed correlations, between all (*n* = 136) parcellated ROIs > Connectivity clusters (*n* = 210), computed using CONN’s data-driven hierarchical clustering > Connectivity networks (*n* = 20), using CONN’s network clustering	> Co-inertia (CIA) analysis to assess the relationship between gut microbiome, serum metabolome and connectivity > Permutation ANOVA to assess the association between BD-related microbial taxa and BD-related region-to-region connectivity, which was thereafter clustered into connectivity clusters (only performed in cases) Multiple testing correction: NA	
	[43]	Dong (2022)	Collection: not reported Sequencing type: 16s rRNA sequencing Sequencing technology: Illumina Hiseq Region: V4 Pipeline: QIIME (v1.9.1) Database: Greengenes (no version) Clustering: OTU Rarified: yes (diversity analysis) Filtering: OTUs with prevalence >10% Transformation: ratio (Prevotella/Bacteroides) (*Prevotella/Bacteroides)*			*> Prevotella*/ *Bacteroides* ratio, based on clustering analysis > No selection for compositional analyses	rs-fMRI Eyes closed	Brainstem and left NAcc, based on significant differences in connectivity between obese and non-obese	> Region-to-region Fisher’s z-transformed correlations, between all (*n* = 430) parcellated ROIs > Network centrality measures (degree strength, betweenness centrality and eigenvector centrality) computed for all parcellated ROIs	> Spearman correlations between brain connections and microbiome measures with significant differences between obese and non-obese groups (only performed in cases) Multiple testing correction: FDR	
	[41]	Labus (2019)	Collection: tube with RNAlater Stabilization Solution Sequencing type: 16s rRNA sequencing Sequencing technology: Roche 454 Region: V5-6 Pipeline: Lotus (v1.32) Database: GreenGenes (v13.8) and RDP II (v11) Clustering: OTU Rarified: no Filtering: genera with >20% prevalence Transformation: relative abundance			Genera in the order of Clostridia, based on ability to regulate host serotonin biosynthesis, including *Clostridium* IV, *Faecalibacterium* and *Oscillibacter* from the family Ruminococcaceae and *Clostridium* XIVa, *Clostridium* XIVb, *Blautia*, *Coprococcus*, *Roseburia* and *Lachnospiraceae* incertae sedis from the Lachnospiraceae family	rs-fMRI Eyes closed	Mid- and posterior insula, somatosensory cortex (S1, S2, M1, M2), basal ganglia (NAcc, caudate, putamen, pallidum) and thalamus, based on findings from earlier study in IBS patients.	Network topological indices of region-to-region Fisher’s z-transformed correlation network (degree strength, betweenness centrality, eigenvector centrality)	> Tripartite network analysis based on Fisher’s z-transformed Spearman’s correlations between abundances, connectivity and gastrointestinal function (covariates: age and sex) > Network difference IBS-HC: Z-test on correlation coefficient difference between groups Multiple testing correction: none	
	[45]	Hong (2021)	Collection: fresh Sequencing type: shotgun metagenomics Sequencing technology: not reported (Illumina) Pipeline: MetaPhlAn2 Database: IGC and KEGG (no versions) Filtering: no Transformation: relative abundance			No selection for compositional analyses	rs-fMRI Eyes closed	Right putamen and left SMA, based on alterations in regional homogeneity established in first part of the analyses.	Seed-to-voxel Fisher’s z-transformed correlations, between ROIs and all other voxels in the brain.	Partial correlation analyses to test association between changes in connectivity and changes in gut microbiota from pre- to post-surgery. Covariates: age and sex Multiple testing correction: FDR	
	[40]	Jacobs (2021)	Collection: fresh Sequencing type: 16s rRNA sequencing Sequencing technology: Illumina Hiseq2500 Region: V4 Pipeline: QIIME (v1.9.1) Database: GreenGenes (v13.5) Clustering: OTU Rarified: yes (diversity analysis) Filtering: not specified Transformation: not specified			No selection for compositional analyses	rs-fMRI Eyes closed DTI		FC: Region-to-region Fisher’s z-transformed correlations, between all (*n* = 248) parcellated ROIs SC: FA and Apparent Diffusion Coefficient in all segmented white matter regions	Partial correlations between changes in connectivity and changes in microbiota composition (only for genera and brain connections with significant CBT-induced change) Covariates: age and sex Multiple testing correction: FDR	Measures collected pre- and 2 weeks post CBT
	[39]	Ahluwalia (2016)	Collection: tube with RNAlater Stabilization Solution Sequencing type: 16s rRNA LH-PCR fingerprinting and multitag pyrosequencing Sequencing technology: ABI 3130xl fluorescent capillary sequencer Region: universal 16s primers Pipeline: custom PERL pipeline Database: RDP Clustering: OTU Rarified: no Transformation: relative abundance Filtering: taxa present in each sample, with relative abundance threshold ≥1%, unclear whether on OTU or genus level			No selection for compositional analyses	DTI	Corpus callosum, internal capsule, inferior and superior longitudinal fasciculi, frontal and posterior white matter, uncinate fasciculi, insula and corticospinal tracts	Mean fractional anisotropy, mean diffusivity, and mean spherical isotropy for each white matter tract of interest.	Correlation analyses between structural connectivity indices and abundance Multiple testing correction: none	

*ACC* anterior cingulate cortex, *AN* auditory network, *CBT* cognitive behavioral therapy, *dACC* dorsal anterior cingulate cortex, *DAN* dorsal attention network, *dlPFC* dorsolateral prefrontal cortex, *DMN* default mode network, *ECN* executive control network, *ESRD* end-stage renal disease, *FA* fractional anisotropy, *FC* functional connectivity, *FCD* functional connectivity density, *FPN* frontoparietal network, *FWE* family wise error, *HC* healthy controls, *IBS* irritable bowel syndrome, *ICA* independent component (analysis), *LefSE* linear discriminant analysis effect size, *M1* primary motor cortex, *M2/SMA* supplementary motor area, *Maaslin2* microbiome multivariable association with linear models, *MDD* major depressive disorder, *NAcc* nucleus accumbens, *OTU* operational taxonomic unit, *PD* phylogenetic diversity, *PLS-DA* partial least-squares discriminant analysis, *RDP* ribosomal database project, *ROI* region of interest, *S1* primary sensory cortex, *S2* secondary sensory cortex, *SC* structural connectivity, *sCCA* sparse canonical correlation analysis, *SMN* sensorimotor network, *SN* salience network, *TPF* temporo-parietal-frontal network, *TSS* total sum scaling, *VAN* ventral attention network, *VN* visual network, *wUF* weighted UniFrac.

### Methodological characteristics (Table 2)

**Fig. 2:**
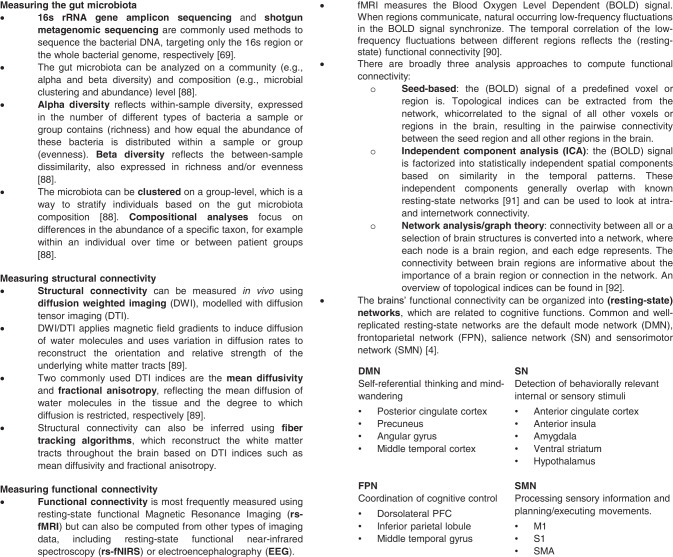
Overview of commonly used techniques. Overview of commonly used techniques and indices to investigate the gut microbiota [69, 87, 88] and functional and structural brain connectivity [89–92].

An overview of the methods and indices used to quantify and analyze the gut microbiota and functional and structural brain connectivity is provided in Fig. 2.

#### Microbiota quantification

The included studies employed various sequencing workflows to estimate the gut microbiota composition. Three studies performed shotgun metagenomic sequencing [36, 38, 45], while all other studies performed 16s rRNA gene sequencing.

##### Microbial diversity

Six out of sixteen studies tested the association between alpha diversity/richness and connectivity [27, 28, 30–33, 35, 36], and one out of sixteen studies assessed beta diversity [35].

##### Microbial composition

Three studies performed microbiota-based clustering [27, 28, 30, 31, 34], and all but two studies assessed microbial abundance. Additional methodological information regarding sample collection and data processing is presented in Table 2.

#### Connectivity quantification

##### Functional connectivity assessment

Fourteen out of sixteen studies assessed functional connectivity, of which twelve used resting-state functional magnetic resonance imaging (rs-fMRI), one used task-based fMRI [33], and one used resting-state functional near-infrared spectroscopy (fNIRS) [36].

##### Structural connectivity assessment

Four out of fourteen studies assessed structural connectivity using diffusion tensor imaging (DTI) [28, 34, 39, 40]. Additional information about the metrics used to compute connectivity, and the a priori selection of brain regions, networks, or white matter tracts for each study is provided in Table 2.

#### Statistical analysis on microbiota-connectivity association

The included studies employed various statistical methods to explore the association between microbiota and brain connectivity, including linear regression/ANCOVA [32, 33, 35, 36, 46], (permutation) ANOVA [27, 28, 38, 44], (partial) correlation [27–31, 34, 39, 40, 43, 45] and tripartite network analysis, based on correlations [41]. Three studies used packages for differential abundance testing, including sparse partial least squares discriminant analysis (sPLS-DA) [34, 44], linear discriminant analysis effect size (LefSE) [36], microbiome multivariable association with linear models (MaAsLin2) [36], and differential gene expression analysis based on the negative binomial distribution (DESeq2) [44]. Finally, linked ICA [37], spatial canonical correlation analysis (sCCA) [33] and Parametric Empirical Bayes (PEB) [33] analysis were employed by one study each.

**Table 3:** Overview of synthesized study findings.

	Ref	First author (year)	Microbiota community - Connectivity associations	Microbiota composition - Connectivity associations^a^	Other
Healthy population *Adults*	[32]	Curtis (2019)	> Number of OTUs positively associated with connectivity between left middle insula and frontal pole and associated with connectivity between right inferior insula and right occipital cortex (no direction). > Association between wUF PC2 and connectivity between left middle insula and cerebellar vermis 9 region (n.s. after Bonferroni); between wUF PC2 and connectivity between right anterior insula and right lingual gyrus; and between left anterior insula and cerebellum lobes 4 and 5 (no direction).	> *Bacteroides* negatively associated with connectivity between left anterior insula and left operculum. > *Prevotella* positively associated with connectivity between left anterior insula and right occipital cortex (n.s. after Bonferroni).	
	[27–29﻿]	Cai (2021)(a)/Zhu (2022)(b)/Zheng (2022) (c) (three publications based on the same study)	Internetwork (a): > Simpson positively correlated with connectivity between pDMN and rFPN (1), pDMN and AN (2), rFPN and DAN (3), rFPN and dSMN (4), rFPN and mVN (5), and between rFPN and IVN. Simpson negatively correlated with aDMN and lFPN (1), ECN and IVN (2), lFPN and rFPN (3), DAN and pVN (4) and between dSMN and pVN. > pDMN-AN and lFNP-rFPN connectivity mediated the relationship between Simpson and sleep quality score. rFPN-mVN connectivity mediated the relationship between Simpson and working memory. DAN-pVN connectivity mediated the relationship between Simpson index and attention. Intranetwork (a): > Ace, Chao and Sobs positively correlated with intranetwork connectivity in the bilateral lateral PFC of the ECN. Negative correlation between Shannon and intranetwork connectivity in the right angular gyrus of the rFPN. Functional (FCD) and structural (FA) connectivity mixing coefficients (b): > Chao positively correlated with FCD of IC4, the FA of IC3 and the FA of IC5. > FCD of IC4 and FA of IC3 mediated the positive association between Chao and attention. Network properties (c): > Structural: Shannon was negatively correlated with normalized clustering coefficient and the small-worldness property (global network properties), and normalized clustering coefficient and the small-worldness property partially mediated the association between Shannon and working memory (3-back accuracy). > Functional: Shannon negatively correlated with the nodal degree centrality of the left and right median cingulate and paracingulate gyri (mediated the association between Shannon and digit span backward), and the nodal efficiency of the left median cingulate and paracingulate gyri. Shannon was also positively correlated with the nodal degree centrality and nodal efficiency of the right inferior temporal gyrus. Simson was negatively correlated with the nodal degree centrality of the right inferior temporal gyrus. > SC-FC coupling: Simpson was negatively associated with the SC-FC coupling of the right inferior occipital gyrus, which partially mediated the association between Simpson and accuracy on the go/no-go task.	Intranetwork (a): > ↑ intranetwork connectivity in the left OFC (part of the lFPN) *Prevotella* and *Ruminococcus* clusters compared to *Bacteroides* cluster. This connection mediated the relationship between clusters and response inhibition. Functional (FCD) and structural (FA) connectivity mixing coefficients (b): > Interaction effect between clusters and sex, with lower FCD and FA IC2 coefficients in the *Prevotella* cluster vs. *Bacteroides* cluster in women, and higher FCD and FA IC2 coefficients in the *Prevotella* cluster vs. *Ruminococcus* cluster in men. Network properties (c): > Structural: nodal degree centrality *Ruminococcus* > *Bacteroides* for the left inferior temporal gyrus, right supramarginal gyrus and right caudate nucleus. *Bacteroides > Ruminococcus* for left and right posterior cingulate gyrus. *Bacteroides* > P*revotella* for the left posterior cingulate gyrus. > Structural: nodal efficiency *Ruminococcus* > *Bacteroides* for the right supramarginal gyrus and left caudate nucleus. *Bacteroides > Prevotella* for the left posterior cingulate gyrus. > Functional: nodal degree centrality *Ruminococcus > Bacteroides* for right anterior cingulate and paracingulate gyri, superior temporal gyrus. > Functional: nodal efficiency *Ruminococcus > Bacteroides* for the right superior temporal gyrus. > Functional: nodal betweenness *Prevotella > Bacteroides* for right angular gyrus (mediated the association between enterotypes and digit span forward); *Bacteroides > Ruminococcus* for right superior temporal gyrus; and *Ruminococcus > Prevotella* for left dorsolateral superior frontal gyrus. > SC-FC coupling: *Ruminococcus* > *Bacteroides* for the left hippocampus and left fusiform gyrus (mediated the association between enterotypes and digit span forward); *Bacteroides > Ruminococcus* for the right anterior cingulate and paracingulate gyri*;* and *Bacteroides > Prevotella* for the left supramarginal gyrus and left medial superior frontal gyrus (mediated the association between enterotypes and digit span backward).	Spatial mapping of ICs (FCD and FA) (b): > IC2 - FCD: Herschl’s gyrus, insula, superior parietal lobule, fusiform gyrus, temporal pole, precuneus, middle cingulate cortex > IC4 - FCD: distributed regions in the lateral PFC, precentral/postcentral gyrus, posterior insula, angular gyrus, lateral occipital cortex, lateral temporal cortex, precuneus, dorsomedial PFC, ventromedial PFC > IC2 - FA: corpus callosum, internal capsule, optic radiation > IC3 - FA: temporal and occipital juxtacortical white matter > IC5 - FA: scattered WM regions across the frontal and parietal lobes
	[33]	Hall (2021)	> Inverse Simpson is associated with inhibitory connectivity from dorsal ACC to anterior insula during threat reversal, but not during threat acquisition. > No associations between B/F-ratio and connectivity.	> *Ruminococcus* associated with dorsal ACC-anterior insula connectivity during threat acquisition and threat reversal. > *Bacteroides* associated with dorsal ACC-anterior insula connectivity during threat acquisition. > *Oscillospira* and *Prevotella* were not associated with connectivity.	
	[34]	Tillisch (2017)		> Ten structural connections could discriminate between *Prevotella*- and *Bacteroides*-high clusters with 66.7% accuracy (*Prevotella* > *Bacteroides*): middle frontal gyrus-central sulcus, amygdala-caudate, ACC-pallidum, fusiform gyrus-inferior temporal gyrus, anterior transverse collateral sulcus-inferior temporal sulcus, inferior temporal sulcus-parallel sulcus, thalamus-pericallosal sulcus, posterior ramus of the lateral sulcus-temporal pole, thalamus-temporal pole, posterior mid cingulate gyrus/sulcus -central sulcus. > All but two structural connections identified by discriminant analysis were positively associated with *Prevotella* relative abundance but not with *Bacteroides* relative abundance.	
	[37]	Kohn (2021)		> *Prevotella-9* was positively associated and *Blautia* was negatively associated with pDMN and ECN intranetwork connectivity. > *Prevotella-9* and *Bacteroides* were negatively associated and *Bifidobacterium* was positively associated with aDMN intranetwork connectivity. > *Prevotella-9*, *Bifidobacterium*, *Faecalibacterium* and genera belonging to *Lachnospiraceae* (f) were positively associated and *Christensenellaceae R-7 group* was negatively associated with FPNs (left and right) intranetwork connectivity. > *Ruminococcus* was positively and *Blautia* was negatively associated with internetwork connectivity between DMN and SN (specifically: dorsal ACC and vlPFC).	
Healthy population *Children*	[35]	Gao (2019)	> Shannon, number of OTUs, Faith’s phylogenetic diversity and Chao were negatively associated with connectivity between mid/forebrain Thalamus and left amygdala, between ACC and right anterior insula and between S2 and IPL.		
	[36]	Kelsey (2021)	> Shannon and Chao (taxonomic) were positively associated with left FPN intranetwork connectivity. > Shannon and Chao (taxonomic) were positively associated with homologous-interhemispheric intranetwork connectivity (n.s. after covariate correction). Homologous interhemispheric connectivity mediated the association between Shannon and Chao and negative emotionality (component of behavioral temperament). > Virulence factor diversity was positively associated with homologous-interhemispheric intranetwork connectivity. Homologous interhemispheric connectivity mediated the association between virulence factor diversity and orienting behaviors.	> ↑ of genus *Clostridium* perfringens with low left DMN connectivity. > ↑ of genus *Clostridium* (sp. disporicum, perfringens, tertium) with high FPN connectivity. > ↑ of species within genera *Enterococcus*, *Collinsella*, *Prevotella*, *Robinsoniella* and *Bacteroides* with high left FPN connectivity; > ↑ of species within genera *Streptococcus* and *Enterococcus* with low left FPN connectivity. > ↑ of *Escherichia* coli with high homologous-interhemispheric network connectivity. > ↑ of *Bifidobacterium* dentium with low homologous-interhemispheric network connectivity. > *Clostridium* associations with FPN (21 taxa) and DMN (2 taxa) were replicated by Maaslin2.	
Disease population *Adu﻿lts*	[44]	Dong (2020)	> No significant differences in Shannon’s index between patients with high and low precuneus-putamen functional connectivity.	> sPLS-DA classifier with ROC of 0.97 for classifying high vs. low precuneus-putamen connectivity based on microbial composition. Thirty ASVs contributed to the first component: *Bacteroides* (g), Lachnospiraceae (f), *Methanobrevibacter* (g), *Alistipes* (g) and *Dorea* (g) were the top five taxa associated with low connectivity. *Anaerostipes* caccae (s), Lachnospiraceae (f), *Anaerostipes* hadrus (s), *Butyricicoccus* desmolans (s) and *Lachnospira* (g) were the top five taxa associated with high connectivity. > 28 taxa associated with precuneus-putamen connectivity. Low connectivity: 26 taxa, of which 5 belonging to *Bacteroides* (g) and 8 to *Ruminococcaceae* (f). High connectivity: unclassified *Ruminococcaceae* s.2 (f) and unclassified CAG-56 (g).	
	[42]	Strandwitz (2019)		> Negative correlation between *Bacteroides* relative abundance and functional connectivity between dlPFC and medial frontal cortex (part of DMN). > No association between *Evtepia* gabavorous and connectivity.	
	[30, 31]	Wang (2019) (a)/Zheng (2020) (b) (two publications based on the same study)	> No significant differences in alpha diversity between ESRD and HC. Therefore, no association analyses performed between alpha diversity and connectivity.	DMN (a): > *Roseburia* (↑ in HC) was positively associated with pDMN-aDMN connectivity, partially mediated by levels of interleukin-6 ( ↑ in ESRD) in ESRD patients. > *Prevotella* (↑ in ESRD) was negatively associated with DMN dissociation (clustering coefficient, local efficiency) in the ESRD group (not reported for the HC group). > *Vogesella* is negatively associated with aDMN-pDMN connectivity. > Normalized clustering coefficient of the DMN is positively associated with *Odoribacter*, *Selenomonas, Schwartzia, Syntrophus* and negatively associated with: *Collinsella, Coprobacillus, Prevotella, Comamonas, Epulopiscium, Heliciobacter*. > Local efficiency of the DMN is negatively associated with *Collinsella, Coprobacillus, Comamonas and Prevotella*. Amygdala (b): > *Roseburia* (↑ in HC) was positively associated with amygdala-IPL connectivity, partially mediated by levels of TNF-α in ESRD patients. > ESDR patients in the *Prevotella*-high cluster had higher connectivity between amygdala-ACC/superior frontal cortex and amygdala-caudate/putamen compared to ESRD patients in the *Bacteroides*-high cluster.	
	[38]	Li (2022)		> Co-inertia analysis shows that the gut microbiota displays global similarity with functional connectivity, although not significantly. > 78.3% of microbial taxa that showed a significant difference between BD and HC were significantly correlated with at least on functional connection (region-to-region*)*. *>* Associations between functional connectivity and the genera (or species within the genera) *Clostridium, Prevotella, Sutterella, Eubacterium, Bacteroides, Enterobacter, Alistipes, Catabacter, Streptococcus, Lachnoclostridium, Parabacteroides, Ruminococcus, Blautia* and *Phascolarctobacterium* were most frequently identified. > The gut microbiota were associated with functional connections distributed throughout the brain, the most consistently in networks centered around the hippocampus, amygdala, thalamus, striatum or inferior temporal gyrus.	
	[43]	Dong (2022)		> Eigenvector centrality of the left NAcc was positively associated with the *Prevotella/Bacteroides* ratio and genus *Eubacterium* abundance, and negatively associated with *Bacteroides* abundance. > Eigenvector centrality of the brainstem was negatively associated with abundance of the genera *Oribacterium*, *Actinomyces* and *Fusobacterium* and family *Gemellaceae*.	
	[41]	Labus (2019)		Associations significantly stronger in HC compared to IBS > *Lachnospiraceae* Incertae Sedis positively correlated with S2 connectivity. > *Coprococcus* negatively correlated with caudate connectivity. > *Clostridium* XIVa associated with Putamen, S1 and NAcc connectivity. > *Clostridium* XIVb correlated with S1, M1 connectivity. > *Clostridium* IV correlated with M1. > *Blautia* positively correlated with S2 and negatively correlated with M1 connectivity. Associations significantly stronger in IBS compared to HC > *Roseburia* mostly negatively correlated with posterior insula and pallidum connectivity. > *Oscillibacter* positively associated with middle and posterior insula.	
	[45]	Hong (2021)		> No associations between pre- to post surgery changes in the gut microbiota composition and changes in putamen or SMA connectivity.	
	[40]	Jacobs (2021)		> In CBT responders: increases in *Bacteroides* and unclassified S24-7 from pre- to post CBT were negatively associated with decreased connectivity between the brainstem and left lateral aspect of the superior temporal gyrus and the right planum temporal (both part of the temporal network). > No significant associations between pre- to post CBT microbial changes and changes in structural connectivity.	
	[39]	Ahluwalia (2016)		> *Porphyromonadaceae* (f) relative abundance was negatively correlated with FA of corpus callosum splenium, right inferior longitudinal fasciculus, posterior internal capsule and posterior white matter (left and right); positively correlated with spherical isotropy of the corpus callosum splenium, right interior longitudinal fasciculus and posterior white matter; positively correlated with MD of corpus callosum genus, corpus callosum splenium, L + R posterior white matter, L + R frontal white matter and the right uncinate fasciculus. > *Prevotellaceae* (f) relative abundance was positively associated with FA of the right posterior white matter. > *Veillonellaceae* (f) was positively correlated with MD in the bilateral anterior internal capsule, corpus callosum splenium, right cingulum, external capsule, posterior internal capsule and right uncinate fasciculus.	

*ACC* anterior cingulate cortex, *aDMN* anterior default mode network, *AN* auditory network, *ASV* amplicon sequence variant, *B/F* Bacteroidetes/Firmicutes, *CBT* cognitive behavioral therapy, *DAN* dorsal attention network, *dlPFC* dorsolateral prefrontal cortex, *ECN* executive control network, *ESRD* end-stage renal disease, *f* family, *FA* fractional anisotropy, *FC* functional connectivity, *FCD* functional connectivity density, *FPN* frontoparietal network, *g* genus, *HC* healthy control, *IBS* irritable bowel syndrome, *IC* independent component, *IPL* inferior parietal lobe, *M1* primary motor cortex, *MD* mean diffusivity, *n.s.* non-significant, *OFC* orbitofrontal cortex, *OTU* operational taxonomic unit, *PC* principal component, *pDMN* posterior default mode network, *PFC* prefrontal cortex, *ROC* receiver operating characteristic, *s* species, *S1* primary somatosensory cortex, SC structural connectivity, *SMA* supplementary motor area, *SMN* sensorimotor network, *sPLS-DA* sparse partial least-squares discriminant analysis, *vlPFC* ventrolateral prefrontal cortex, *VN* visual network, *WM* white matter, *wUF* weighted UniFrac.

^a^Taxonomic findings are on genus level, unless otherwise specified.

### Synthesized results (Table 3)

Results on the microbiota level are discussed first, starting with findings in alpha- and beta diversity, followed by findings in microbial composition (clusters and abundance). Next, results on the brain connectivity level are discussed, starting with findings in functional connectivity, followed by findings in structural connectivity. Finally, the specificity of the association between the microbiota and connectivity is discussed. A detailed summary of the findings per individual study is available in the Supplementary Materials.

#### Gut microbiome

##### Microbial diversity

**Fig. 3:**
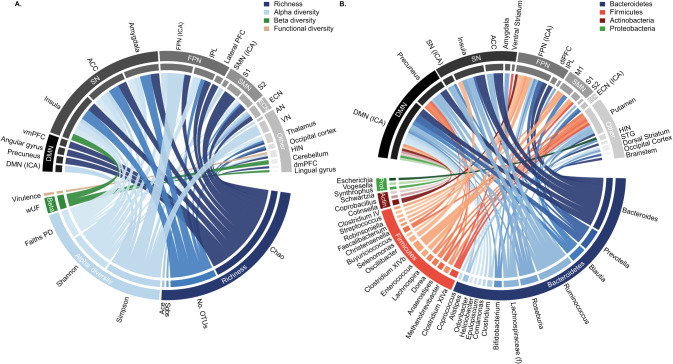
Associations between the gut microbiota and functional connectivity. Graphic summary of the reported associations between microbial diversity and functional connectivity (**A**), and between microbial abundances and functional connectivity (**B**). Each connection in the chord diagram reflects a reported association between the abundance of that genus or diversity measure and functional connectivity. Functional connectivity is aggregated to the network level, including intra-/ and internetwork connectivity and connectivity of individual constituents of a particular network, to aid visualization. Of note, the graphical overview displays the absolute number of reported associations, skewing it towards genera and connectivity networks that are studied more frequently (a consequence of the selection bias in the reported studies). ACC anterior cingulate cortex, DMN default mode network, ECN executive control network, FPN frontoparietal network, SMN sensorimotor network, SN salience network.

Six out of sixteen studies assessed microbial richness and/or alpha diversity in association with brain connectivity [27–29, 32, 33, 35, 36, 44]. Four out of six studies assessed a measure of microbial richness (number of OTUs, Sobs, Ace and/or Chao), all of which report an association with at least one brain connection or network [27–29, 32, 35, 36]. All six studies assessed a measure of alpha diversity (Shannon or Simpson), of which four studies reported an association with at least one brain connection or network [27–29, 33, 35, 36] (Fig. 3A, Supplementary Table 1). Only one study assessed a measure of beta diversity (weighted UniFrac), and reported an association with functional brain connectivity [32]. As there is only one study assessing this, it does not warrant interpreting at this stage (Supplementary Table 1). A comprehensive overview of the results per study can be found in Table 3.

##### Microbial composition: clustering

Three out of sixteen studies performed clustering of the microbiome to obtain microbiome clusters/enterotypes. Of those, two studies identified two clusters: one high in *Bacteroides* and one high in *Prevotella* [30, 31, 34]. One study identified three clusters: a *Bacteroides*-high, *Prevotella*-high, and *Ruminococcus*-high cluster [27–29]. All three studies reported at least one association with connectivity, either functional [27–31] or structural [28, 29, 34] (see Table 3). Given the low number of studies, variability in number of clusters, and uncertainty about what the clusters consist of, it remains difficult to interpret the findings and draw conclusions about potential consistency (Supplementary Table 1).

##### Microbial composition: abundance

Thirteen out of sixteen studies assessed microbial abundance on the genus level, all of which report at least one significant association with functional and/or structural brain connectivity (for an overview, see Supplementary Table 1). The genera *Bacteroides*, reported in nine out of eleven studies [32, 33, 36–38, 40, 42–44], and *Prevotella*, reported in six out of ten studies [30–32, 34, 36–38], were most consistently associated with brain connectivity. Additionally, genera within the order *Clostridiales* were also repeatedly reported in association with brain connectivity. Within this order, the genus *Ruminococcus* was most consistently reported (in four out of seven studies [30, 31, 33, 37, 44]), followed by *Blautia* (in three out of five studies [37, 38, 41]). Other studies meeting the criteria for consistency include *Collinsella* (three out of five studies [30, 36, 38]), *Enterococcus* [36, 38], and *Alistipes* [38, 44] (each reported in two out of five studies) and *Bifidobacterium* (two out of three studies [36, 37]). These genera were associated with regions distributed throughout the brain, both on a region-to-region and network level. A comprehensive overview of the results per study can be found in Table 3.

#### Brain connectivity

##### Functional connectivity

Fourteen out of sixteen studies assessed the association between functional connectivity and the gut microbiota, either employing a seed-based (i.e., region-to-region or region-to-voxel) or ICA-based (i.e. resting-state networks) approach. All functional connections, seed-based and ICA-based were aggregated to a network level to improve interpretability (Supplementary Table 2). Salience network connectivity was consistently reported in association with the gut microbiome (nine out of ten studies). Particularly the connectivity of the anterior cingulate cortex (ACC) [27–31, 33, 35, 37, 38] and insula [27–29, 32, 33, 35, 38, 41] (each reported in six out of eight studies) were frequently reported. Additionally, amygdala connectivity (three out of six studies [30, 31, 35, 38]) and ICA-based salience network connectivity (two out of three studies [37, 38]) met the criterion of consistency on the brain connectivity level. The default mode network (DMN) was reported in association with the gut microbiota in seven out of nine studies: six out of six studies reported associations between ICA-based DMN-connectivity and the gut microbiota [27–31, 36–38, 42], and associations with the precuneus were reported in three out of six studies [27–29, 38, 44]. Finally, the frontoparietal network (FPN) was consistently associated with the gut microbiota (reported in seven out of nine studies). Particularly, ICA-based FPN-connectivity (four out of four studies [27–29, 36–38]) and connectivity of the dorsolateral prefrontal cortex (three out of six studies [27–29, 38, 42]) and inferior parietal lobe (two out of four studies [30, 31, 35]) were reported with higher frequency. Finally, regions within the sensorimotor network were reported in four out of seven studies (no specific regions within this network) [27–29, 35, 38, 41] and the superior temporal gyrus was associated with the gut microbiota in three out of six studies [27–29, 38, 40]. These connectivity networks were exhibiting associations with a wide range of diversity indices and genera abundances. A comprehensive overview of the findings per study can be found in Table 3 and Supplementary Table 2.

##### Structural connectivity

Three out of sixteen studies assessed structural connectivity, all of which reported at least one association with gut microbiota diversity or composition [28, 29, 34, 39]. However, the small number of studies and the variability in how structural connectivity was quantified and labeled, precludes meaningful interpretation at this time (Table 3, Supplementary Table 1).

#### Specificity of the microbiota-connectivity associations

Despite observing recurring patterns in findings on the gut microbiota and brain connectivity level, patterns are not evident in the association between microbiota and connectivity. None of the microbiota-connectivity associations were reported in at least fifty percent of the studies, thus not meeting the consistency criterion. Instead, diversity indices and microbial abundances were associated with a widespread set of brain regions and networks without any discernible pattern emerging. That is, the association between the gut microbiota and brain connectivity was non-specific (Fig. 3, Supplementary Tables 1 and 2).

### Quality assessment

A majority of the studies were rated as ‘Fair’ (*n* = 9), four studies were rated as ‘Poor’ and only three studies were rated as ‘Good’. The primary source of bias was related to incomplete reporting of the methods, including a lack of detail in the description of the recruitment procedure, microbiota data handling, and description of the used statistical methods. Additionally, about a third of the studies did not correct for the effects of key confounders (i.e., age and sex). The quality assessment and explanatory notes per study are shown in Supplementary Table 6.

## Discussion

### Summary

A qualitative systematic synthesis of the available study findings shows associations between the gut microbiota and brain connectivity. On the microbiota level, a majority of the studies reported an association between microbial richness or diversity and connectivity. In terms of genus abundance, the genera *Bacteroides*, *Prevotella*, *Ruminococcus*, *Blautia*, and *Collinsella* were reported with the highest consistency, followed by the genera *Enterococcus*, *Alistipes*, and *Bifidobacterium*. For functional brain connectivity, the highest level of consistency was found for the DMN, FPN, and salience network (particularly the insula and ACC). There were too few studies assessing microbial clusters or structural connectivity to draw definite conclusions at this time. Moreover, although some microbial genera or brain networks were reported with higher frequency, there was no specificity in the association between the gut microbiome and brain connectivity, and a majority of the findings, both in microbiota and brain, were inconsistent and poorly or not replicated across studies. Since some of the included studies did not provide information on the direction of the associations, and as the interpretation of the direction depends on the specific brain connection/network involved, we decided not to consider the directionality in our synthesis. Consequently, the conclusion is limited to the observation of a potential association between the gut microbiota and brain connectivity.

Finally, the studies differed in their target population, focusing on a range of different diseases. Considering the large methodological differences between studies as well as a lack of direct comparisons between the case and control participants, is presently not possible to draw conclusions about differences in the microbiota-connectivity associations between cases and controls or between different disease populations.

Below we will first discuss the findings in relation to neurocognitive functioning and possible functional mechanisms. Finally, we will address study aspects that can explain inconsistencies in the findings, and which need to be considered to further advance the field.

### Neurocognitive functions associated with microbiota

The current findings suggest an involvement of brain networks involved in emotion-related cognition and executive functioning. The association between the gut microbiota and emotion-related functioning is a recurring topic in gut-brain research. Several of the included studies found associations between the microbiota composition and levels of anxiety, depression, or negative affect, measured using questionnaires [30, 31, 34]. Moreover, several of the brain structures associated with the gut microbiota have a putative emotion-related function. For example, the insula, whose connectivity was associated with alpha diversity [32, 33, 35] and with the abundance of the genera *Roseburia* and *Bacteroides* [28, 32, 41], is involved in socio-emotional processing, and insular brain damage can result in apathy and anxiety [47]. Moreover, the insula is involved in interoception, both emotional and visceral [8, 9]. The amygdala, whose connectivity was associated with alpha diversity and *Roseburia* abundance [31, 35], is one of the brain’s main emotion-processing structures, which is implicated in, among others, autism and anxiety [48]. Finally, the DMN, whose connectivity was associated with microbial diversity and the abundance of a wide range of bacterial genera (Fig. 3), is involved in mind-wandering and conceptualization of emotions [49]. Moreover, DMN connectivity is altered in depression, a disorder characterized by impairments in emotion regulation [50]. Interestingly, Kelsey and colleagues [36] reported that stronger intranetwork connectivity of the homologous-interhemispheric network in newborns mediated the association between higher alpha diversity and behavioral temperament, behavior that is predictive of anxiety and depression in adulthood [51].

Another set of findings suggests that the gut microbiota may be associated with executive functioning. Cai and colleagues [27] reported that higher internetwork connectivity between the FPN and visual networks and lower internetwork connectivity between the dorsal attention, and visual networks mediated the association between higher alpha diversity and better working memory and attention. In addition, the association between *Prevotella-*, *Bacteroides-*, and *Ruminococcus*-high clusters and response inhibition was mediated by connectivity of the orbitofrontal cortex (part of the FPN), a region involved in (emotional) decision making [52]. Specifically, individuals in the *Prevotella* and *Ruminococcus*-high clusters exhibited stronger orbitofrontal connectivity, which was associated with poorer response inhibition. Consistent with these findings, several functional networks associated with gut microbiota diversity are involved in executive functioning. For example, the FPN (or its individual constituents) was associated with the abundance of, among others, the genera *Prevotella*, *Bacteroides*, and *Blautia* [36, 37, 46]. This network is mainly involved in executive control, encapsulating processes related to response inhibition, attention, and working memory [6, 53].

Altogether, the associations between the gut microbiota and the brain, described in this review, indirectly link the gut microbiota with processes related to emotion and executive functioning through brain connectivity patterns. However, in the absence of direct, empirically tested, associations with cognition and behavior, this should be interpreted with caution. These speculations should be validated using, for example, larger-scale mediation analyses to disentangle the intermediate role of brain connectivity in the association between the gut microbiota and behavior.

### Microbial function and functional pathways

Up to now, studies mostly report taxonomic findings, which are not appropriate to deduce functional pathways. Nevertheless, we can still leverage the findings from previous studies to speculate about potential mechanisms, which can be used as hypothesis-generating ideas as the field moves towards more function-focused microbiome research. Based on the taxonomic findings, one possible communication pathway would be through the production of short-chain fatty acids (SCFAs; e.g., acetate, propionate, and butyrate). For example, species within the genera *Prevotella* and *Bacteroides*, whose abundances on a genus level were associated with connectivity of a widely distributed set of brain regions and networks (Fig. 3), are described as main propionate and acetate-producing species [54]. Moreover, species in the genus *Blautia*, whose abundance on a genus level was associated with the connectivity of sensorimotor regions as well as network connectivity of the DMN and executive control network, are described to have propionate-producing properties [54]. Finally, species in the genus *Roseburia*, whose abundance on a genus level was associated with insular, amygdala, and DMN connectivity, are among the main butyrate-producing species. Among others, SCFAs possess the ability to modulate immune activation (reviewed in [55]). In line with this, the association between *Roseburia* abundance and functional connectivity of the DMN and amygdala, reported by Wang and colleagues [30] and Zheng and colleagues [31], was mediated by levels of the pro-inflammatory cytokines interleukin-6 and tumor necrosis factor alpha, suggesting a role for the immune signaling pathway in the microbiota-brain connectivity communication, possibly through the production of butyrate. The involvement of this pathway may go beyond the gut, as there is preliminary evidence for a role of the immune signaling pathway in the association between functional connectivity and the oral microbiome as well [56]. Other ways through which gut-synthesized SCFAs may modulate gut-brain communication, and hereby brain connectivity, could be by positively or negatively affecting the integrity of the intestinal and blood-brain barriers [55, 57, 58], or by traveling directly through the blood-brain barrier to the CNS [59–61], although – similar to the above-suggested role of the SCFAs – evidence directly supporting this is yet lacking.

The gut microbiota may also affect gut-brain communication through the production of neurotransmitters and their precursors [46]. For example, species within the genera *Bacteroides* and *Bifidobacterium* and species within genera *Ruminococcus* and *Blautia*, whose abundances were associated with connectivity in a widespread set of brain regions and networks, are major modulators of GABA and serotonin availability in the gut [62–64]. There are several proposed pathways through which gut-derived neuroactive compounds can affect the brain. For example, there is evidence showing that both GABA and serotonin have immunomodulatory properties [65, 66], and both GABA and serotonin receptors have been located on vagal afferents to the CNS, proposing a role for the vagal signaling pathway [67, 68]. However, in the absence of direct evidence, the involvement of these pathways remains hypothetical.

Altogether, based on the taxonomic findings we can speculate about potential mechanisms. However, most taxonomic findings are reported on a genus level due to the constraints of 16s rRNA sequencing. A certain genus, and even a single species within a genus, can contribute to multiple metabolite pathways. As such, our speculations should be verified through pathway analysis, preferably using higher resolution metagenomics data coupled with, when possible, bacterial culture-functional studies.

### Recommendations for future studies

The current review identified several links between microbial genera and brain connectivity, but there is low specificity in the associations between the gut and brain. Additionally, despite identifying some recurring patterns, a majority of the findings, both in microbiota and brain, were inconsistent and poorly replicated across studies. This may suggest a complex multifaceted relationship between microbial composition and brain connectivity, but it is likely that inconsistencies are at least partially methodology-driven. The limitations identified in this review underscore the need to harmonize the methodological approaches currently applied to microbiome research and (functional) brain connectivity analysis. In the following section, we will discuss important study aspects that may explain inconsistencies in the findings, and which need to be considered to further advance the field.

#### Methodological comparability

The number of studies investigating the microbiota-gut-brain axis is rapidly growing, and in recent years there have been major technological advances in the field. Nevertheless, there is no golden standard on how to collect, process, and analyze microbiome data. As a result, there is high inter-study variability in laboratory processing (e.g., variable region of the 16S rRNA gene and sequencing platform), pre-processing (e.g., taxonomic database, processing pipeline and prevalence filtering, and data transformation) and statistical analysis approach. The number of observed taxa and statistical outcomes can change considerably depending on the collection, storage, bioinformatic pipeline, and statistical test used [69–72]. Therefore, to make reliable between-study comparisons it is essential to harmonize the methodological approach. To achieve this, researchers should follow standardized reporting guidelines, such as the STORMS checklist [25], on how the microbiome data was processed, follow standardized processing pipelines where possible, apply consistent and appropriate statistical approaches for analysis (e.g., accounting for the zero-inflated and compositional nature of the data), and report findings (including effect-sizes) in a complete and transparent manner.

While reducing the multiple comparison problem, additional sources of inter-study variability come from a priori selection of microbial genera and brain regions (e.g., seed-based resting-state analyses). Some of these genera – for example *Prevotella* and *Bacteroides* – or brain networks – for example the DMN – are of common interest in studies focusing on the gut-brain communication, increasing the likelihood that the effects are observed or amplified as a result of selection bias. Moreover, a priori selection, both in taxonomy and brain regions, hinders meaningful comparison between study findings. At the current stage of the field, using a data-driven approach to study the association between the gut microbiota and brain connectivity will help with the identification of patterns of associations in the data without biasing the findings towards pre-existing assumptions. However, data-driven approaches have a higher risk of identifying spurious associations, and findings may be more challenging to interpret. Therefore, once consistent findings have been established using data-driven approaches, the field could move towards a hypothesis-driven framework that offers clearer research questions and facilitates the interpretation of the findings.

#### Data integration and multivariate approaches

Data analysis should better acknowledge the inherent complexities of both the gut microbiota and brain connectivity data, as this will lead to better integration of the two domains. Both the gut microbiota and the brain are complex systems, characterized by intricate relationships and interconnections [73, 74]. At the current time, most studies investigating the association between the gut microbiota and brain connectivity do so using simple bivariate association analyses, even though this is likely an oversimplification of the existing association. As a result, information concealed in the relationship within and between multiple variables (i.e., interaction effects) is lost [75]. Although several studies opted for a multivariate approach for one of the two domains (i.e., differential abundance testing for the microbiome, and ICA or sparse canonical correlation analysis for brain connectivity), only one study used an integrative multivariate approach, integrating microbiota and connectivity data [37]. The authors identified several multivariate associations between microbial clusters and functional network connectivity.

Future studies should make an effort to integrate data from the gut microbiota and brain connectivity. Possible approaches are the Linked Independent Component Analysis (as applied in ref. [37]) or co-inertia analysis (as applied in ref. [38]), which allow for simultaneous factorization of data from different domains. Moreover, the systems biology approach focuses on multivariate interactions in biological systems rather than exploring each modality in parallel [76]. There are already different systems biology software programs available [77]. However, they are mainly utilized to integrate the so-called ‘omics’ techniques and, so far, there is little contribution of macroscale neurobiological measures. Nevertheless, the field of human connectomics was already proposed as an extension of systems biology to macroscale neuroscience [78–80] and could provide insight into the complex microbiota-connectivity interactions.

Another advantage of integrating more (-omics) domains would be improved mechanistic insight into the human gut-brain axis. There have been numerous studies on possible mechanisms underlying gut-brain communication, both preclinical and in humans (reviewed in e.g. ref. [81]). Usually, mechanisms are studied on a molecular level, without assessing how such processes would affect the brain on a larger scale. To date, only Wang and colleagues [30] and Zheng and colleagues [31] explored the mediation effect of immune activation as an underlying mechanism in the gut-brain connectivity association. Although we can speculate about underlying mechanisms based on taxonomic findings, it would be more informative to directly integrate mechanistic data (potentially) related to brain connectivity. For example, metagenomics can complement 16s rRNA sequencing to provide information about the functional potential of the present microbial taxa, metabolomics could provide information about bacterial metabolites that are present in the gut (e.g., SCFAs), and inflammatory markers in the blood could be investigated to provide information about immune activation.

#### Confounders

There is currently no agreement on which factors that affect the gut should be considered and corrected for in statistical analyses. This is also reflected in the way studies correct for confounders: approximately two-thirds consider age and sex as key confounders. Recent antibiotic use is used as an exclusion criterion by three-fourths of the studies, whereas only one study corrects for smoking. Surprisingly, none of the studies correct for ethnicity [82], medication use [83], or diet [17, 84], even though several studies have demonstrated their effects on the gut microbiome and/or brain. To better understand how these factors affect the association between microbiota and brain connectivity, we recommend that future studies conduct sensitivity analyses. This will – besides establishing the robustness of the findings – help to identify the (often environmental) factors that influence the association between the gut microbiota and brain connectivity and potentially inform the development of future treatment strategies.

#### Study characteristics and group comparisons

Currently, studies assessing the association between the gut microbiota and brain connectivity include a wide range of target populations, from healthy individuals to various disease populations. While these studies could provide valuable information about disease pathophysiology, the field lacks a reference to which associations in disease populations can be compared. Investigating the microbiota-connectivity associations in large population cohorts can provide such references and aid in the interpretation of study findings. Moreover, with a case-control study design, it is possible to test differences in the microbiota-connectivity association between groups. However, only one study has performed such analyses [41], with other studies only focusing on the microbiota-connectivity association in cases. Performing such comparative analyses will help in clarifying whether there are differences in the associations between different disease populations, and improve our ability to draw meaningful conclusions about group differences.

Once such methodological issues are resolved, it would be valuable to have longitudinal microbiota-connectivity association studies, for example in developing children or elderly individuals, to see how microbial development covaries with neurodevelopment, whether microbiota-connectivity associations vary over time and how this could be related to neurodevelopmental or neurodegenerative disorders.

#### Association not causation

Most studies discussed here were observational, from which it is not possible to infer causality nor directionality. To advance research on the human MGBA, specifically for the development of gut microbiota-targeting approaches in the treatment of brain-related disorders, it is important to establish causality and directionality. Assessing directionality and causality in human trials remains a costly challenge, in which randomized controlled trials in combination with appropriate statistical models such as mediation or Mendelian randomization can assist [85, 86]. For example, results from mediation analyses point towards gut-to-brain signaling, showing that immune activation (one of the MGBA signaling pathways) partially mediated the association between microbial abundance and functional brain connectivity [27, 36]. Such studies should assess if and how an intervention affects the gut microbiota or brain connectivity, which microbes are causally involved, if intervention-induced changes in the microbiota result in altered brain connectivity, and – importantly – whether this is also reflected in altered cognition and behavior.

## Conclusion

In this systematic review, we identified several genera as well as brain regions and networks that were repeatedly associated in microbiota-brain connectivity analyses, showing the potential of employing brain connectivity measures to gain insight into gut-brain communication. At the same time, there is still limited evidence for specificity in the microbiota-brain connectivity association. Current methodological limitations, including high inter-study variability in methodology and target population, small sample sizes, the a priori selection of microbial genera and brain regions of interest, and the statistical approaches used, introduce bias and thus contribute to the inconsistent findings. We know the gut-brain communication is multidimensional, and therefore a more systematic and harmonized methodology, which acknowledges this complexity, is key to further unraveling how the gut and brain communicate. To enhance comparability and replicability, future research should focus on further standardizing processing pipelines and employ data-driven multivariate analysis approaches. Moreover, interventional studies can help to clarify the causality and directionality of the reported findings.

### Supplementary information

Supplementary Tables

Study descriptions

PRISMA checklist
