# Supplementary material for: A systematic review exploring the association between the human gut microbiota and brain connectivity in health and disease
Source: Mol Psychiatry. 2023 Jul 21;28(12):5037–61. doi: 10.1038/s41380-023-02146-4 (PMC11041764; doi:10.1038/s41380-023-02146-4)
Supplement: Supplementary file 2 — Study descriptions [file 41380_2023_2146_MOESM2_ESM.docx]

**Supplementary materials: study summary**

Herein we provide a summary of the findings of each study, individually. First, studies investigating healthy participants are discussed, covering studies in adults first, followed by studies in children. Second, studies in a disease population are discussed. In each section, results on microbial diversity will be discussed first, followed by results on microbial composition (microbial clustering and abundance).

- - 1. *Healthy population: adults*

*Microbial diversity*

Three studies have assessed functional connectivity and microbial alpha and/or beta diversity in a sample of healthy adults. Curtis and colleagues [1] assessed the association between seed-based functional connectivity of the insula and alpha and beta diversity, and reported an association between microbial richness (number of OTUs) and the functional connectivity between the insula and occipital cortex. The direction of the association was not reported. Regarding beta diversity, associations between weighted UniFrac distance and the connectivity between the insula and the operculum (non-significant after Bonferroni correction), lingual gyrus and cerebellum were reported.

A study conducted by Cai and colleagues**,** Zhu and colleagues, and Zhang and colleagues [2–4] yielded three publications. In the first publication [2] the authors reported a positive association between microbial richness (Sobs, Ace, Chao) and intra-network connectivity of the executive control network and between alpha diversity (Shannon) and intra-network connectivity of the FPN. Moreover, alpha diversity (Simpson) was associated with inter-network connectivity between networks related to executive control, default mode and sensorimotor systems. In the second publication [3], the authors associated multimodal independent components, based on among others functional connectivity density and fractional anisotropy, with richness and diversity. Using this approach, microbial richness (Chao) was found associated with a functional and structural connections distributed throughout the brain. The third publication [4] focused on functional and structural network organization, identifying associations between alpha diversity (Shannon, Simpson), global network properties of the structural network, and regional network properties of the functional network, mainly within the cingulate and temporal cortex. Additionally, there were associations between alpha diversity (Simpson) and the coupling between structural and functional connectivity of occipital regions.

Finally, Hall and colleagues [5] investigated the association between alpha diversity (Simpson) and the directed functional connectivity between the dorsal anterior cingulate cortex (dACC) and anterior insula during threat processing (threat acquisition and reversal), and revealed that there was a positive association between alpha diversity and the inhibitory connectivity from the dACC to the anterior insula connectivity during threat reversal, but not during threat acquisition.

*Microbial composition: clustering*

Two studies performed microbiota-based clustering to associate the gut microbiota with functional and/or structural brain connectivity in healthy adults. The three publications based on the study by Cai and colleagues**,** Zhu and colleagues and Zhang and colleagues [2–4] describe three microbiota-based clusters characterized by high Prevotella, high Bacteroides, or high Ruminococcus abundance. In the first publication [2], associating these clusters to fourteen ICA-based resting-state networks showed that the participants within the *Prevotella-* and *Ruminococcus-clusters* had higher intra-network connectivity of the left FPN compared to participants in the *Bacteroides-*cluster. In the second publication [3], the authors used a multimodal independent components and identified sex-dependent differences between microbial clusters in functional and structural connectivity strength. In the third publication [4], differences between clusters were reported in both functional and structural network organization, both in terms of global and regional network properties. Moreover, there were differences between clusters in the coupling between functional and structural connectivity in a distributed set of brain regions.

Finally, Tillisch and colleagues [6] identified two clusters, one characterized by high *Prevotella* abundance (containing seven participants), and one characterized by *Bacteroides* abundance (containing 33 participants), and investigated which structural connections could best discriminate between the two clusters. Discriminant analysis revealed ten structural connections, distributed throughout the brain, whose strength could discriminate between the clusters with 66.7% accuracy.

*Microbial composition: abundance*

Four studies assessed the association between microbial abundance and functional (three studies) or structural (one study) brain connectivity in healthy adults. Curtis and colleagues [1]**,** assessing the genera *Prevotella* and *Bacteroides*, reported a negative association between *Bacteroides* relative abundance and the functional connectivity between the anterior insula and the operculum, and a positive association between *Prevotella* relative abundance and the functional connectivity between the anterior insula and the occipital cortex, the latter being non-significant after Bonferroni correction.

Hall and colleagues [5] assessed the association between the abundances of *Bacteroides*, *Prevotella*, *Oscillospira*, and *Ruminococcus* and directed threat-related functional connectivity, revealing an association between *Bacteroides* abundance and dACC-anterior insula connectivity during threat acquisition. Additionally, *Ruminococcus* abundance was associated with dACC-anterior insula connectivity during threat acquisition and threat reversal. For threat reversal, this association could be attributed to a positive association between *Rumicococcus* abundance and connectivity from the anterior insula to the dACC.

Kohn and colleagues [7] performed multivariate linked ICA and identified four independent components with contribution from both the gut microbiota and functional network connectivity. First, *Prevotella* abundance was positively and *Blautia* abundance was negatively associated with the intra-network connectivity of the posterior DMN and executive control network. Second, *Prevotella* and *Bacteroides* abundance were negatively, and *Bifidobacterium* abundance was positively associated with intra-network connectivity of the anterior DMN. Third, the abundance of *Bifidobacterium*, *Faecalibacterium* and genera belonging to the *Lachnospiracaceae* family were positively and *Christensenellacea_R-7* was negatively associated with the intra-network connectivity of the FPNs. Fourth, *Ruminococcus* abundance was positively and *Blautia* abundance was negatively associated with the internetwork connectivity between the DMN and SN.

Finally, Tillisch and colleagues [6] assessed the association between *Prevotella* and *Bacteroides* relative abundance and the ten structural connections with the highest explanatory value in differentiating between *Prevotella* and *Bacteroides* microbiota clusters, as discussed above. *Prevotella* relative abundance was associated with all but two of the ten structural connections, while *Bacteroides* relative abundance was not associated with any of the connections.

- - 1. *Healthy population: children*

Microbial diversity

Two studies assessed functional brain connectivity and microbial alpha diversity in a sample of healthy children. Gao and colleagues [8] investigated one-year-old infants and reported a negative association between microbial richness (number of OTUs, Chao) and diversity (Shannon, Faith’s PD) and the connectivity between the amygdala and thalamic regions, and between the anterior cingulate cortex and anterior insula. Additionally, there was a positive association between alpha diversity/richness and the connectivity between the supplementary motor area and the inferior parietal lobule.

Kelsey and colleagues [9] assessed taxonomic and functional alpha diversity in one-month-old infants, revealing a positive association between taxonomic alpha diversity/richness (Chao, Shannon) and frontoparietal and homologous-interhemispheric intranetwork connectivity. Additionally, there was a positive association between virulence factor diversity (i.e., diversity of bacteria producing molecules associated with disease) and intranetwork connectivity of the homologous-interhemispheric network.

*Microbial composition: abundance*

Kelsey and colleagues [9] explored the association between functional brain connectivity and gut microbiota composition in one-month-old infants, revealing enrichment of species within the genus *Clostridium* in infants with high frontoparietal and low default mode intranetwork connectivity. Moreover, high frontoparietal intranetwork connectivity was characterized by enrichment of species *Enterococcus* faecalis*,* *Collinsella* (unclassified species), *Prevotella* copri, *Robinsoniella* peoriensis and *Bacteroides* caccae, whereas *Enterococcus* (unclassified species) and *Streptococcus* salivarius were enriched in low left frontoparietal intranetwork connectivity. Finally, high homologous-interhemispheric intranetwork connectivity was characterized by enriched *Escherichia* coli, and low homologous-interhemispheric intranetwork connectivity by enriched *Bifidobacterium* dentium.

- - 1. *Disease population: adults*

*Microbial diversity*

The association between alpha diversity and (functional) brain connectivity in a disease population was only assessed by one study. Dong and colleagues [10] examined the link between alpha diversity (Shannon) and functional connectivity between the precuneus and putamen in obese patients undergoing a laparoscopic sleeve gastrectomy. Data pooled from before and after surgery showed no differences in alpha diversity between patients with high and low precuneus-putamen connectivity.

*Microbial composition: abundance*

Nine studies assessed brain connectivity and microbial composition in a disease population, of which two were case-only studies with one timepoint, four were case-controlled with a group of healthy adults, and three were case-only using a longitudinal design. Eight out of nine studies assessed functional connectivity and two studies assessed structural connectivity. Strandwitz and colleagues [11] investigated the association between relative abundance of the genus *Bacteroides* and the functional connectivity between the left dorsolateral prefrontal cortex (dlPFC) and the DMN in patients with major depressive disorder, and reported that *Bacteroides* relative abundance was negatively correlated with dlPFC-DMN connectivity.

A study performed by Wang and colleagues and Zheng and colleagues [12,13] in patients with end-stage renal disease yielded two publications. In the first publication [12] *the authors assessed the association between intranetwork DMN connectivity and microbial composition, and reported a positive association between Roseburia relative abundance and connectivity between the anterior and posterior DMN. This association was partially mediated* by levels of the pro-inflammatory cytokine *interleukin-6. The relative abundances of Colinsella, Coprobacillus, Comamonas, Epulopiscium, Heliciobacter, Odoribacter, Prevotella, Schwartzia, Selenomonas, Syntrophus* and *Vogesella,* were also associated with DMN network organization (clustering coefficient and local efficiency), but these associations were not mediated by inflammation markers. In the second publication [13], the authors focused on the association between microbial composition and seed-based amygdala functional connectivity, and identified an association between Roseburia absolute abundance and the connectivity between the amygdala and the inferior parietal lobule. This association was partially mediated by levels of the pro-inflammatory cytokine tumor necrosis factor alpha.

Li and colleagues [14] assessed the gut microbiota composition and functional connectivity in patients with bipolar disorder. A total of 78% of microbial taxa that were differentially abundant in cases compared to controls were also associated with at least one functional connection in the brain. The genera *Clostridium, Prevotella* and *Suterella* were most frequently reported, together with functional networks centered around the hippocampus, amygdala, thalamus, striatum, and inferior temporal gyrus.

Dong and colleagues [15] investigated the gut microbiota composition and functional brain network properties in individuals with obesity. The authors reported associations between the eigenvector centrality, a metric reflecting the influence of one region in a network, of the nucleus accumbens and the ratio between Prevotella and Bacteroides abundance, as well as Eubacterium abundance. Additionally, the eigenvector centrality of the brainstem was associated with abundance of the genera *Oribacterium*, *Actinomyces* and *Fusobacterium*.

Labus and colleagues [16] performed a case-control study to explore the association between relative abundance of genera in the order Clostridia and resting-state functional connectivity of sensorimotor brain regions in patients with irritable bowel syndrome (IBS) and healthy adults. Using tripartite network analysis, significant differences in the associations between sensorimotor brain regions and *Clostridium* (*XIVa* and *XIVb)*, *Coprococcus,* and an unclassified Lachnospiraceae genus were identified between cases and controls: associations were observed in healthy adults but were largely absent in patients with IBS. One exception is the *Roseburia* genus: in patients with IBS, there were multiple, mostly negative, associations between this genus and connectivity, whereas such associations were absent in healthy adults.

Dong and colleagues [10] examined the association between gut microbiota composition and the connectivity between the precuneus and putamen in obese patients undergoing laparoscopic sleeve gastrectomy. Discriminant analysis showed that the microbial composition could discriminate between participants with a high and low precuneus-putamen connectivity with an ROC of 0.97. *Bacteroides, Methanobrevibacter, Alistipes* and *Dorea* were enriched in participants with high precuneus-putamen connectivity, and *Anerostipes*, *Lachnospira* and *Butyricococcus* were enriched in participants with a low precuneus-putamen connectivity.

Hong and colleagues [17] performed a longitudinal study to examine the association between changes in gut microbiota composition and changes in seed-based connectivity of the putamen and supplementary motor area from pre- to post vertical sleeve gastrectomy in obese patients. The surgery did induce changes in functional connectivity and microbial composition, but the changes were not associated.

Jacobs and colleagues [18] performed a longitudinal study to investigate how cognitive behavioral therapy (CBT) affected the gut microbiota composition and functional and structural connectivity in responding and non-responding patients with irritable bowel syndrome, and how such CBT-induced changes were associated. The authors reported increases in *Bacteroides* and unclassified S24-7 relative abundance in CBT responders compared to non-responders. Those changes were furthermore associated with decreased functional connectivity between the brainstem and regions within the superior temporal gyrus. CBT also induced changes in structural connectivity, but those were not associated with changes in the microbial composition.

Finally, Ahluwalia and colleagues [19] investigated the association between microbial composition and structural connectivity strength of the brain’s major white matter tracts in patients with cirrhosis, revealing associations between the abundance of the bacterial families *Porphyromonadaeae*, *Prevotellaceae* and *Veillonellaceae* and structural connections distributed throughout the brain.

**References**

1 . Curtis K, Stewart CJ, Robinson M, Molfese DL, Gosnell SN, Kosten TR et al. Insular resting state functional connectivity is associated with gut microbiota diversity. *Eur J Neurosci* 2019; **50**: 2446–2452.

2 . Cai H, Wang C, Qian Y, Zhang S, Zhang C, Zhao W et al. Large-scale functional network connectivity mediate the associations of gut microbiota with sleep quality and executive functions. *Hum Brain Mapp* 2021; **42**: 3088–3101.

3 . Zhu J, Wang C, Qian Y, Cai H, Zhang S, Zhang C et al. Multimodal neuroimaging fusion biomarkers mediate the association between gut microbiota and cognition. *Prog Neuro-Psychopharmacology Biol Psychiatry* 2022; **113**: 110468.

4 . Zhang S, Xu X, Li Q, Chen J, Liu S, Zhao W et al. Brain Network Topology and Structural–Functional Connectivity Coupling Mediate the Association Between Gut Microbiota and Cognition. *Front Neurosci* 2022; **16**: 1–17.

5 . Hall C V., Harrison BJ, Iyer KK, Savage HS, Zakrzewski M, Simms LA et al. Microbiota links to neural dynamics supporting threat processing. *Hum Brain Mapp* 2022; **43**: 733–749.

6 . Tillisch K, Mayer EA, Gupta A, Gill Z, Brazeilles R, Le Nevé B et al. Brain Structure and Response to Emotional Stimuli as Related to Gut Microbial Profiles in Healthy Women. *Psychosom Med* 2017; **79**: 905–913.

7 . Kohn N, Szopinska-Tokov J, Llera Arenas A, Beckmann CF, Arias-Vasquez A, Aarts E. Multivariate associative patterns between the gut microbiota and large-scale brain network connectivity. *Gut Microbes* 2021; **13**: 2006586.

8 . Gao W, Salzwedel AP, Carlson AL, Xia K, Azcarate-Peril MA, Styner MA et al. Gut microbiome and brain functional connectivity in infants-a preliminary study focusing on the amygdala. *Psychopharmacology (Berl)* 2019; **236**: 1641–1651.

9 . Kelsey CM, Prescott S, McCulloch JA, Trinchieri G, Valladares TL, Dreisbach C et al. Gut microbiota composition is associated with newborn functional brain connectivity and behavioral temperament. *Brain Behav Immun* 2021; **91**: 472–486.

10 . Dong TS, Gupta A, Jacobs JP, Lagishetty V, Gallagher E, Bhatt RR et al. Improvement in Uncontrolled Eating Behavior after Laparoscopic Sleeve Gastrectomy Is Associated with Alterations in the Brain–Gut–Microbiome Axis in Obese Women. *Nutrients* 2020; **12**: 1–16.

11 . Strandwitz P, Kim KH, Terekhova D, Liu JK, Sharma A, Levering J et al. GABA-modulating bacteria of the human gut microbiota. *Nat Microbiol* 2018; **4**: 396–403.

12 . Wang YF, Zheng LJ, Liu Y, Ye YB, Luo S, Lu GM et al. The gut microbiota-inflammation-brain axis in end-stage renal disease: Perspectives from default mode network. *Theranostics* 2019. doi:10.7150/thno.35387.

13 . Zheng LJ, Lin L, Zhong J, Zhang Z, Ye YB, Zhang XY et al. Gut dysbiosis-influence on amygdala-based functional activity in patients with end stage renal disease: a preliminary study. *Brain Imaging Behav* 2020; **14**: 2731–2744.

14 . Li Z, Lai J, Zhang P, Ding J, Jiang J, Liu C et al. Multi-omics analyses of serum metabolome, gut microbiome and brain function reveal dysregulated microbiota-gut-brain axis in bipolar depression. *Mol Psychiatry* 2022; **27**: 1–13.

15 . Dong TS, Guan M, Mayer EA, Stains J, Liu C, Vora P et al. Obesity is associated with a distinct brain-gut microbiome signature that connects Prevotella and Bacteroides to the brain’s reward center. *Gut Microbes* 2022; **14**: 1–17.

16 . Labus JS, Osadchiy V, Hsiao EY, Tap J, Derrien M, Gupta A et al. Evidence for an association of gut microbial Clostridia with brain functional connectivity and gastrointestinal sensorimotor function in patients with irritable bowel syndrome, based on tripartite network analysis. *Microbiome* 2019; **7**: 1–15.

17 . Hong J, Bo T, Xi L, Xu X, He N, Zhan Y et al. Reversal of Functional Brain Activity Related to Gut Microbiome and Hormones After VSG Surgery in Patients With Obesity. *J Clin Endocrinol Metab* 2021; **106**: 3619–3633.

18 . Jacobs JP, Gupta A, Bhatt RR, Brawer J, Gao K, Tillisch K et al. Cognitive behavioral therapy for irritable bowel syndrome induces bidirectional alterations in the brain-gut-microbiome axis associated with gastrointestinal symptom improvement. *Microbiome* 2021; **9**: 1–14.

19 . Ahluwalia V, Betrapally NS, Hylemon PB, White MB, Gillevet PM, Unser AB et al. Impaired Gut-Liver-Brain Axis in Patients with Cirrhosis. *Sci Rep* 2016; **6**: 1–11.
